# A Treatise on the Theory and Practice of Obstetrics

**Published:** 1873-04

**Authors:** 


*A Treatise on the Theory and Practice of Obstetrics.* By Wm. H. Byford, A. M., M. D. Second Edition, thoroughly revised. New York: Wm. Wood & Co., 1873. Buffalo: H. H. Otis.

The Second Edition of Prof. Byford's work, comes to us in many respects highly improved over the former. Much of the work has been rewritten and the entire book has been thoroughly revised. The author has made several additions, which will add much to its merit as a work on Obstetrics. Those who were possessed of the first edition of Prof. Byford's work, although not agreeing with some of its teachings, regarded it as an excellent practical treatise on Obstetrics. Not claiming to discuss the finer points, or to settle the disputed methods of procedeure he gave to the profession a plain practical work.

As it has been for some time known to the profession, it is unnecessary for us to discuss its contents or to point out its beauties or defects; it simply remains to be said that the present edition is improved in many respects, and is entirely new in others. We have no hesitation in recommending it to the student or practitioner as a work of great excellence, unsurpassed as a practical guide and book of reference.

